# Encoding of forelimb forces by corticospinal tract activity in the rat

**DOI:** 10.3389/fnins.2014.00062

**Published:** 2014-05-01

**Authors:** Yi Guo, Richard A. Foulds, Sergei V. Adamovich, Mesut Sahin

**Affiliations:** Department of Biomedical Engineering, New Jersey Institute of TechnologyNewark, NJ, USA

**Keywords:** brain computer interfaces, corticospinal tract, time-frequency analysis

## Abstract

In search of a solution to the long standing problems encountered in traditional brain computer interfaces (BCI), the lateral descending tracts of the spinal cord present an alternative site for taping into the volitional motor signals. Due to the convergence of the cortical outputs into a final common pathway in the descending tracts of the spinal cord, neural interfaces with the spinal cord can potentially acquire signals richer with volitional information in a smaller anatomical region. The main objective of this study was to evaluate the feasibility of extracting motor control signals from the corticospinal tract (CST) of the rat spinal cord. Flexible substrate, multi-electrode arrays (MEA) were implanted in the CST of rats trained for a lever pressing task. This novel use of flexible substrate MEAs allowed recording of CST activity in behaving animals for up to three weeks with the current implantation technique. Time-frequency and principal component analyses (PCA) were applied to the neural signals to reconstruct isometric forelimb forces. Computed regression coefficients were then used to predict isometric forces in additional trials. The correlation between measured and predicted forces in the vertical direction averaged across six animals was 0.67 and *R*^2^ value was 0.44. Force regression in the horizontal directions was less successful, possibly due to the small amplitude of forces. Neural signals above and near the high gamma band made the largest contributions to prediction of forces. The results of this study support the feasibility of a spinal cord computer interface (SCCI) for generation of command signals in paralyzed individuals.

## Introduction

Severe injuries at the cervical spinal cord can result in quadriplegia due to extensive paralysis of the body below shoulders. Brain-computer interfacing (BCI) is a technique to substitute for the lost command signals in these severely paralyzed individuals, using neural signals recorded from the brain. Brain-computer interfaces attempt to “read” the volitional information from various cerebral cortices, primarily those involved in planning and execution of the motor function. In the invasive versions, a large number of microelectrodes implanted in the brain parenchyma record single spike activity of local neurons to extract the volitional information. However, three decades of research has repeatedly concluded that stable recording of individual cell activities with microelectrodes have many technical challenges. The most significant problem is the layer of activated astrocytes that forms around the recording electrode (Biran et al., 2005) and makes it very difficult to follow single spikes over an extended period of time (Perge et al., 2013). Attempts have also been made to characterize the local field potentials and cortical surface recordings with non-penetrating electrodes as a source of volitional information (Schalk et al., 2008; Kubanek et al., 2009).

If we look at the big picture, the final pathway for all the motor control information processed in the brain is the descending tracts of the spinal cord before the signals reach the skeletal muscle. The corticospinal tract (CST) and the rubrospinal tract (RST) together make up the lateral descending system that controls the muscles of the extremities in all the mammals (Iwaniuk and Whishaw, 2000). These two tracts work synergistically (Whishaw and Gorny, 1996; Whishaw et al., 1998), although the relative importance of each may be different in different species. For instance, the magnocellular portion of the red nucleus, where the RST originates (Murray and Gurule, 1979), is larger, thus suggesting a greater role in rodents than in primates (ten Donkelaar, 1988). Unilateral lesioning of the medullary pyramid in rats impaired rotatory movements in the contralateral arm including limb aiming, pronation and supination but spared limb advancement, digit opening, arpeggio and grasping (Whishaw et al., 1998). The red nucleus lesions, in addition to producing similar impairments in rotatory movements, also attenuated arpeggio. Both lesions affected both proximal and distal musculature, however, even after combined lesions the rats were able to advance the limb, grasp food and withdraw the limb (Whishaw et al., 1998), suggesting that some components of skilled limb use are supported by descending neural pathways or spinal cord circuits other than the crossed RST or CST. Whishaw et al. (1998) concluded that rats with pyramidal tract lesions were more impaired in limb guidance than rats with red nucleus lesions, however the relative contributions of each tract were reversed in the control of the wrist and digits. In support of the importance of the CST in the rat, another study showed that the supination of the hand, while reaching for a vertical bar, was lost after a contralateral pyradoctomy (Carmel et al., 2010). Thus, the current research collectively suggests that both CST and RST are recruited in rats synergistically in the control of the forelimbs in a complementary way although the relative importance of each may be different depending on the behavioral context.

The number of fiber counts in the medullary pyramid varies between 73,000 and 150,000 in the rat [depending on whether light or electron microscopy is used (Leenen et al., 1985, 1989; Gorgels, 1990)]. Most of the fibers are slow conducting with diameters less than 1 μm with the largest ones around 3.7 μm (Dunkerley and Duncan, 1969). Being located in the most ventral side of the dorsal column of the rat spinal cord, the fine fibers of the CST can readily be contrasted with the large fibers of the more superficially placed ascending pathways of the dorsal column. The fast fibers reaching up to the speed of 19 m/s (Mediratta and Nicoll, 1983), and presumably the largest ones, are very small in number in the rat CST (Casale et al., 1988).

The nature of the control information descending via the CST is not known; perhaps due to technical difficulties in recording neural signals directly from the tract in experimental animals. Instead, indirect observations are made though micro stimulation of the motor cortex in animals as well as transcranial magnetic stimulation in human subjects where the modulatory effect of CST is seen on the corresponding muscles. Micro stimulation of the primary motor cortex in rhesus macaques generated both facilitatory and suppression effects in both flexor and extensor muscles of the distal and proximal forearm (Park et al., 2004); which is a response presumably mediated through the CST. Transcranial magnetic stimulation of the supplementary and primary motor areas during isometric static hand force task produced EMG effects with similar amplitude and latencies recorded from the intrinsic hand muscles, suggesting that both cortical areas effectively control the spinal cord excitability (Entakli et al., 2013). Indeed, many areas of the neocortex send projections to the spinal cord through the CST. It is interesting to note that complete ablation of the sensorimotor cortex led to the loss of only about 50% of the axons in the medullary pyramid in rats, suggesting that remaining 50% of the myelinated axons come from areas other than the sensorimotor cortex.

Another method of gleaning information about the CST function during behavior is to correlate the single cell activity recorded from a brain region or scalp EEG to the muscle EMG signals. Frequency-domain analysis in humans (Conway et al., 1995) and monkeys (Baker et al., 1997) has shown that isometric muscle contractions at submaximal voluntary force levels are accompanied by synchrony in the beta band between the motor cortex and the EMG signals. This synchronization diminishes during dynamic muscle contractions and is replaced by a higher frequency band around 30–45 Hz (Omlor et al., 2007). Other groups have shown greater contributions in the high-gamma band, with electrocortigograms recorded from the primary motor or premotor cortices, to the upper extremity muscle activities in monkeys (Shin et al., 2012; Nishimura et al., 2013). The post-spike facilitation studies in awake monkeys demonstrated that the tonic discharges related to the static limb torque were more prominent in the corticomotoneurons (CM), cortical neurons that synapse directly on the spinal motor neurons, in contrast to the rubromotoneuronal cells (Lemon et al., 1986; Cheney et al., 1988). [The evidence on existence of CM connections in the rat is controversial (Liang et al., 1991; Alstermark et al., 2004)].

As an alternative approach to BCIs, the lateral descending tracts of the spinal cord may be a potential site for taping into volitional motor signals. Due to the convergence of the cortical outputs into a final common pathway in the descending tracts of the spinal cord, neural interfaces with the spinal cord have a potential of being more compact than BCIs. In light of what we know about the CST, we assert that multi-electrode recordings made in this tract will be able to extract volitional motor information in behaving animals. As in the cortical approaches, the mechanical stability of the recording electrodes in the spinal cord is crucial for those source weights to be stable over time. In our previous work, recordings from the RST were found to be stable in signal amplitudes, but cross validation of regression coefficients for the forelimb kinematics between multiple trials was not successful (Prasad and Sahin, 2006a,b). The poor reproducibility of the regression coefficients was most likely caused by mechanical instability of the electrode interface, the wire and penetrating array electrodes. We then decided to test a flexible substrate, planar electrode array for this application. An electrode assembly with a stainless steel supporting frame was developed to restrict the movement of the array in the cord and extend the lifetime of the interconnecting ribbon cable that runs to the external connector. The well-defined positions of the contacts in the array also permitted sampling of the tract more uniformly than the wire electrodes. The level of success in predicting the forelimb isometric forces using the spinal signals was investigated. Partial results were published as a conference paper (Guo et al., 2013).

## Materials and methods

### Electrode implant

**Figure 1:**
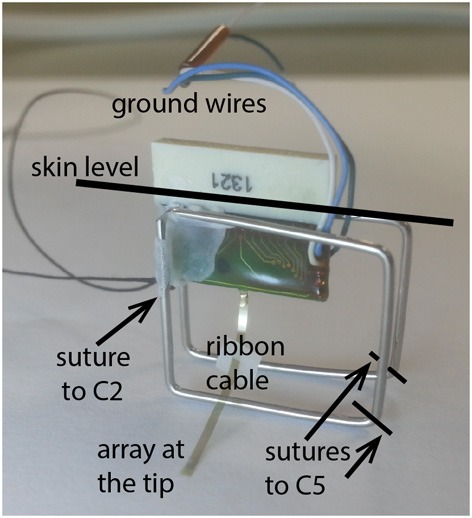
**A photo of the implanted electrode/connector assembly.** The electrode assembly was firmly fixed over the cervical spinal column by suturing the stainless steel wire frame to vertebral bones of C2 on the rostral and C5 on the distal side. The ground wires were placed over the spinal cord as a reference for neural recordings.

Polyimide substrate electrode arrays were custom-designed for this study (NeuroNexus, MI). The array consisted of 4 × 8 arrangement of 32 gold contacts with 15 μm diameter and 80 μm spacing. Every other column of contacts was offset with respect to the neighboring columns by 40 μm to sample the CST cross sectional area more uniformly (see Figure 9). The array had a width of about 650 μm and a thickness of 12 μm. A 2 × 2 mm PDMS sheet (127 μm thick) was attached like a collar around the MEA ribbon cable exactly 1300 μm from the tip (Figure 1). This attachment allowed precise control of penetration depth and enhanced the mechanical stability of the array by keeping it in vertical orientation in the cord.

**Figure 2:**
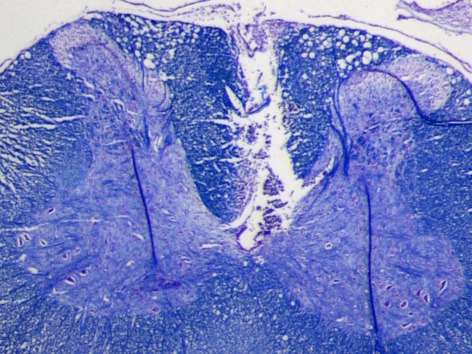
**Luxol fast blue stained transverse section of a cervical spinal cord from one of the rats.** The lesion demarcates where the electrode array was implanted in the dorsal white matter. Because the tissue around was well attached to the substrate during three weeks of implantation time, it was ripped and stayed on the electrode during the explantation. Scale bar is 0.5 mm.

A dorsal laminectomy was performed on C3–C4 segments under ketamine/xylazine anesthesia (80 and 12 mg/kg). The modified electrode array was inserted into the dorsal column mid-sagittally with its contact side facing the preferred hand at C4. The point of entry was the posterior median sulcus of the cord and adjacent to the dorsal vein. The array was pushed into the median septum gently after making a small cut with a #11 blade into the pia matter. A sweet spot exists between the two halves of the spinal cord where electrode penetration did not require much force. The CST occupies a region in the most ventral portion of the dorsal column extending from a depth of 1000–1300 μm measured from the pial surface in the cervical cord. The supporting PDMS attachment was slid under the dura after electrode insertion and a small amount of cyanoacrylate was applied to the ribbon cable where it passes through the dura. A histological slide in Figure 2 depicts the track left behind by the array in the dorsal column after explantation.

The stainless steel wireframe (Ø 0.75 mm) shown in Figure 1 held the electrode assembly in place. The wireframe was tied to the spinous process through a hole made into the C2 vertebra on the rostral side and to the C5 on the caudal end with 6.0 silk sutures. The plastic connector was fixed to the frame with dental acrylic at a height that allowed some slack in the ribbon cable to reduce tension and hence the chronic trauma to the neural tissue. The reference electrode was placed on the dura next to the electrode ribbon cable and glued in place. The neck muscles and the skin were closed in layers around the connector using fine sutures. The plastic connector was protruding ~5 mm through the skin opening. The gap around the connector was sealed with further dental acrylic, which also housed the nuts for anchoring the multi-channel wireless neural amplifier.

### Animal training

**Figure 3:**
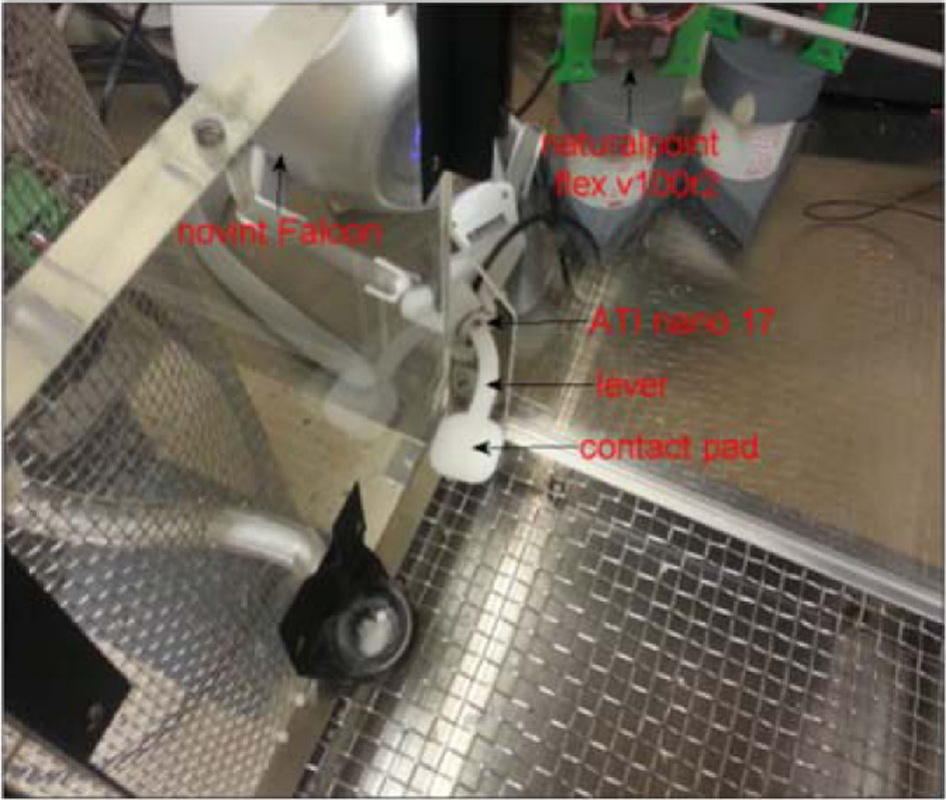
**A photo of the Falcon haptic manipulator with the force sensor and custom-design lever installed into a transparent training box.** The picture shows the corner of the box from inside where a window was opened and the lever arm was brought in through the window. The rats were trained to push down on the contact pad with their preferred hand. The lever is switched to the opposite side of the box for a left-handed animal.

Six Long Evans rats (350–450 g) were used in this study. Food restricted rats were placed in a cage with a lever attached to a computer controlled haptic (with force feedback) device (Falcon, Novint Technologies, NM; see Figure 3). The lever was initially programmed to trigger release of 20 mg sugar pellets on contact. Once the animal became familiar with the lever, the displacement required to trigger food reward was increased incrementally overtime to −14 or 18 mm. The animals used both hands initially to press the lever in most cases. The lever position was switched to the corner of the box on the preferred hand side of the rat after this initial period of training. The animals then learned to do the task with their preferred hand. Behavioral training took 1–2 weeks prior to the implant surgery.

### Data collection

The haptic device (Falcon, Novint) produced 3D positional information at a rate of 1000 samples/s. A force/torque sensor (Nano 17, ATI systems), integrated into the lever, measured the 3D forces applied to the contact pad at the tip of the lever by the rat's arm (Figure 3). Three dimensional force and position information were recorded from the lever. Of interest in the current study was the isometric portion following the lever press. The lever starts moving about 150 ms before the isometric portion begins. All data channels are backlogged continuously for 2000 ms until the lever press is detected. The isometric state, shown as the highlighted portion in the traces (see Figure 5), is achieved with a one-way spring-damper with high impedance to create a virtual boundary by the computer controlled haptic interface. The isometric interval ends when the rat lifts the hand from the lever to move toward the food reward at the end of the trial. The onset and offset time points of the isometric period are decided based on the vertical force being larger than a threshold (~0.11 N).

Neural signals were amplified by a 31 channel wireless system (W32, Triangle Biosystems) before they were digitized at 16 kHz simultaneously with the force data. Infrared cameras generated video logs at 10 fps for quality assessment of the behavioral task. All data streams were synchronized using custom C++ code.

Trained animals performed sessions of 50–200 lever presses (trials) per day until they lost interest in the sugar pellets. Trials with movement artifacts or aberrant postures were excluded from the analysis. Only one day of good data, i.e., one session, was found in each rat after removing all the trials with such defects, which precluded the investigation of multi-day applicability of regression coefficients.

### Time-frequency analysis

**Figure 4:**
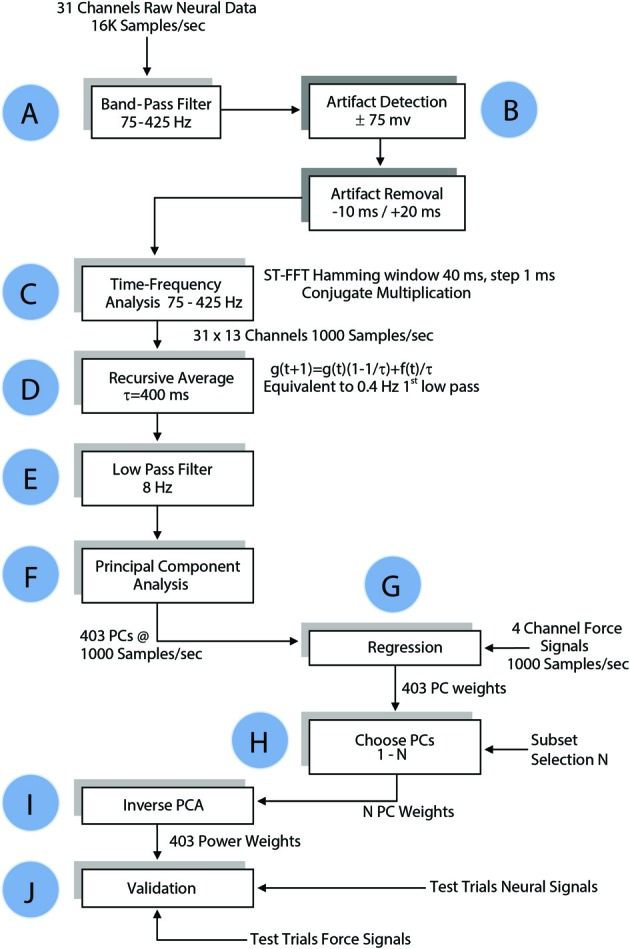
**Flow chart of data processing algorithm for regressing the forelimb force in a single axis.** The same algorithm was applied to all three dimensions of the force in Cartesian coordinates and to the absolute value of the force. See Methods for details.

Neural signals were filtered in both directions in time (to cancel phase delay) using a 3rd order Butterworth band-pass filter at 75–425 Hz (Step A, Figure 4). Any component that exceeded ±75 mV (gain = 800) in the filtered signals was considered an artifact, and upon detection 30 ms of the signal (10 ms preceding it and 20 ms after) was substituted with zeros to remove it (Step B). Power spectral density was computed for each channel of filtered neural signals within a 40 ms moving window that shifted in 1 ms steps (Step C) using short time Fourier transform (STFT), which produced one Fourier coefficient per 25 Hz (total of 13 coefficients within 75–425 Hz band). Time signals representing the power variations in 25 Hz frequency bands were generated by taking the absolute value of FFT coefficients in this moving time window. A total of 403 channels of neural power signals were formed from 31 channels of neural signals (31 neural channels × 13 frequency components for each channel) in each trial. A recursive averaging filter with a 400 ms time constant was applied first to account for the observed low-pass effect of the musculoskeletal system (Step D). A second filter with a sharper transition band (3rd order Butterworth low-pass filter, fc = 8 Hz) was applied to both the neural signals and the forearm forces in both directions in time to eliminate any phase lag (Step E). The force components above 8 Hz were considered as artifacts that could come from mechanical shaking of the lever.

### Principal component decomposition

The principal components (PCs) were computed and sorted in a descending order of variance. The PCs were grouped and regressed against each of the 3-axes of the measured force as well as the magnitude (ABS) of the force vector (Step G in Figure 4). Regression weights of PCs in each group were pre-multiplied with pseudo inverse of corresponding columns of PCA score to generate weights for power signals, as follows:

Let *A* be the transformation matrix from principle components *T* back to power signals *X*:

(1) [T]×[A]=[X]

(2) [T]=[X]/[A]

Forces can be estimated using PCs or power signals. Here *Z* contains regression coefficients of principle components and *W* is the coefficients of power signals *X*:

(3) [F]=[X]×[W]=[T]×[Z]=[X]/[A]×[Z]

Therefore weights of the power signal can be calculated from weights of principle components as follows.

(4) [W]=[I]/[A]×[Z]

*I* is the identity matrix. Matrix division is used because *A* may not be invertible.

### Regression and cross-validation

Approximately one third of the trials in each session were held back for testing and the remaining trials were used for computation of the regression coefficients (training set). The test set data underwent the same time-frequency analysis as the training set. The weights for the power signals corresponding to each set of PCs calculated in the training set were applied to the test set (Steps I and J, Figure 4). The number of PCs in the group was increased incrementally to search for the best regression coefficients (Step H). Goodness of fit was measured by both the coefficient of correlation (*R*) and the coefficient of determination, *R*^2^ (Equation 5), for the entire session with the isometric force episodes taken from each trial and concatenated as a single episode. The weights that generated the best fit to the force in the training set were tested in the test set.

(5) $$R^{2}=1-\frac{\sum_{t}{\left(F-\hat{F}\right)}^{2}}{\sum_{t}{\left(F-\bar{F}\right)}^{2}}$$

where the nominator is the sum of squares of prediction errors and the denominator is the variance of the measured force.

## Results

**Figure 5:**
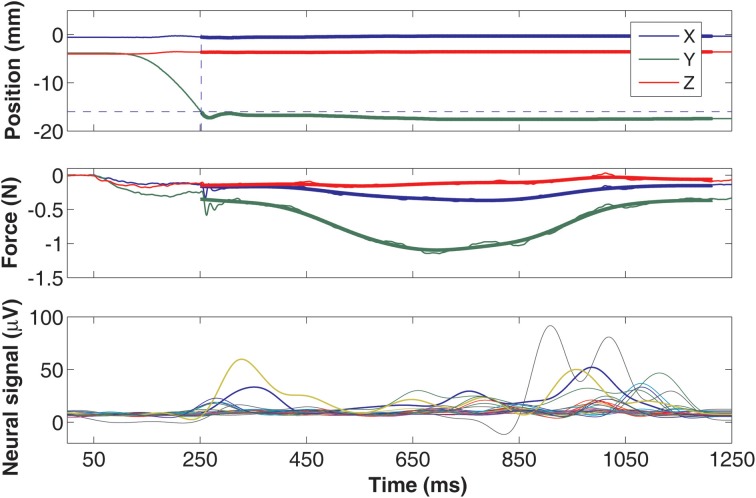
**Plots of position (top) and force traces (middle) from a typical lever press trial (*Y* in green is vertical, *X* in blue and *Z* in red are horizontal components).** The position of the lever is moved from its initial position and reaches the target level (dashed horizontal line) at the dashed vertical line. A threshold force value of 0.11 N was used to determine the starting and ending points of the isometric region. The forces from the isometric portion of the trial used in regression analysis are filtered by an 8 Hz low-pass and plotted as thick lines. The bottom plot shows the rectified-filtered version of all the neural channels.

Three dimensional force and position information recorded from the lever is plotted in Figure 5 in a typical trial. The isometric state is shown as the highlighted portion in the traces. The isometric vertical force (*Y* in green) is the largest as expected for the lever pressing behavior. Nonetheless, small forces are recorded in the side-to-side (*X* in blue) and back-and-forth (*Z* in red) directions as well. The magnitude of the combined force vector (ABS) resembles that of the vertical force since it is the largest component. On the bottom plot, the rectified-filtered versions of the neural signals show different patterns in each channel, indicating spatially selective recordings of the neural sources via different electrode contacts. The neural channels contain signals that are moving at faster rates than the force traces, particularly with very few or no components that co-vary with the overall trajectory of the forces. This suggests that the spinal cord circuitry and the musculoskeletal system together may primarily be working as an integrator/low-pass filter on the descending control signals.

### Optimizing the regression coefficients

**Figure 6:**
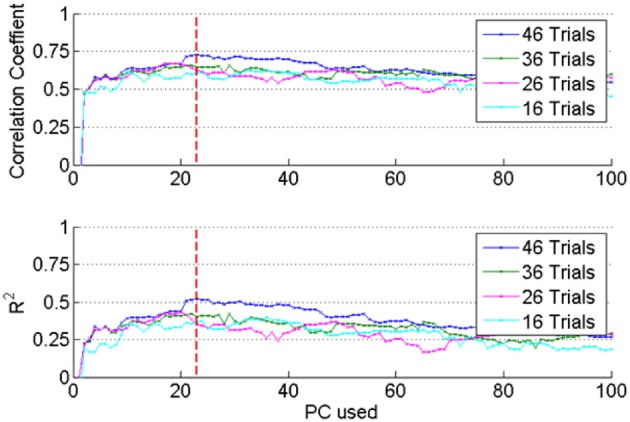
**The optimum regression coefficients were determined by increasing the number of PCs and the number of trials included into the analysis from a session. Top plot**: correlation (*R*), **bottom**: *R*^2^ values obtained by applying the regression coefficients to the test set. Both *R* and *R*^2^ increased with increasing number of PCs up to the dashed vertical line. This procedure prevented over-fitting the data in the training set.

As mentioned above, the number of PCs was varied to search for the best force prediction in each session. Correlation and *R*^2^ values increased (not monotonously but) steadily as additional PCs were included in the regression until a point of diminishing returns (vertical dash line in Figure 6). Adding more PCs over-fit the training set and yielded smaller correlation and *R*^2^ values in the test set, which means that the set of PCs corresponding to the dashed line produced the best possible reconstruction. Furthermore, inclusion of additional trials into the set (e.g., compare the plots for 16 vs. 46 trials in Figure 6) improved the prediction when the optimal set of PCs is used. This further suggested that the reconstruction did not select features that over-fit the training data.

### Reconstruction of forelimb forces

**Figure 7:**
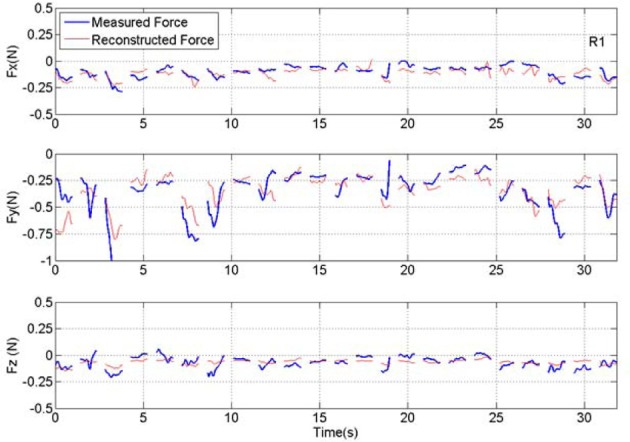
**Plot of measured and reconstructed forces in all three directions in a set of 23 test trials from rat #1 (R1).** Measured force is shown in blue and the force reconstructed from the neural signal is shown in red. Discontinuities in the plots separate the isometric force segments taken from different trials. The correlation values are 0.65, 0.73, and 0.52 for *X*, *Y*, and *Z* directions respectively. The *R*^2^ numbers are 0.35, 0.52, and 0.26 in the same order.

Forelimb forces in all three directions were reconstructed by applying the best coefficients to the test set (Figure 7). Prediction was more effective for the absolute magnitude (not shown) and the vertical force than the other two directions in this and other animals, as indicated by the *R* and *R*^2^ measures (Table 1). In general, the reconstruction algorithm was more successful in predicting the average force amplitude than the rapidly changing components of the forces in each trial. Coefficients of correlation between reconstructed and measured forces for individual trials (rightmost columns in Table 1) were not as high as the overall value for groups of trials in a training or test set. This is because the correlation coefficient for the entire test set accounts for the baseline changes from trial to trial but the correlation for an individual trial removes the baseline and only looks for the resemblance between phasic components of the predicted and actual force profiles within a trial. Thus, the *R* values mostly represent the success in reconstructing the baseline level changes of the forces across multiple trials, although the phasic components are also reproduced in some of the trials.

**Table 1:** **Group statistics of test trials**.

**RAT**	**TRAIN COUNT**	**TEST COUNT**	**TEST Lengths (ms)**		**TEST R**				**TEST R2**				**TEST R by Trial**	
			**MEAN**	**STD**	***X***	***Y***	***Z***	**ABS**	***X***	***Y***	***Z***	**ABS**	**MEAN**	**STD**
1	46	23	905	111	0.65	0.73	0.52	0.72	0.35	0.52	0.26	0.52	0.24	0.56
2	38	18	1015	575	0.60	0.64	0.53	0.64	0.36	0.39	0.24	0.38	0.20	0.65
3	34	18	1923	758	0.56	0.62	0.51	0.60	0.30	0.36	0.25	0.35	0.39	0.42
4	51	26	2433	89	0.59	0.67	0.43	0.66	0.35	0.44	0.17	0.42	0.63	0.25
5	22	12	818	276	0.24	0.58	0.33	0.53	0.05	0.33	0.10	0.28	0.36	0.46
6	81	39	1260	686	0.69	0.77	0.49	0.78	0.46	0.59	0.22	0.60	0.51	0.35
Mean	45.33	22.67			0.56	0.67	0.47	0.66	0.31	0.44	0.21	0.43		
STD	20.16	9.33			0.16	0.07	0.08	0.09	0.14	0.10	0.06	0.12		
Weighted					0.60	0.69	0.48	0.68	0.35	0.47	0.21	0.46		

Correlation (R) and R^2^ statistics of regression in the test set in each rat of the study. Middle columns show the statistics when all the trials in the session are concatenated into one long trial for regression. The last two columns contain mean ± STD of correlations calculated for each trial separately, while the regression was performed again on the concatenated version.

### Frequency contributions

**Figure 8:**
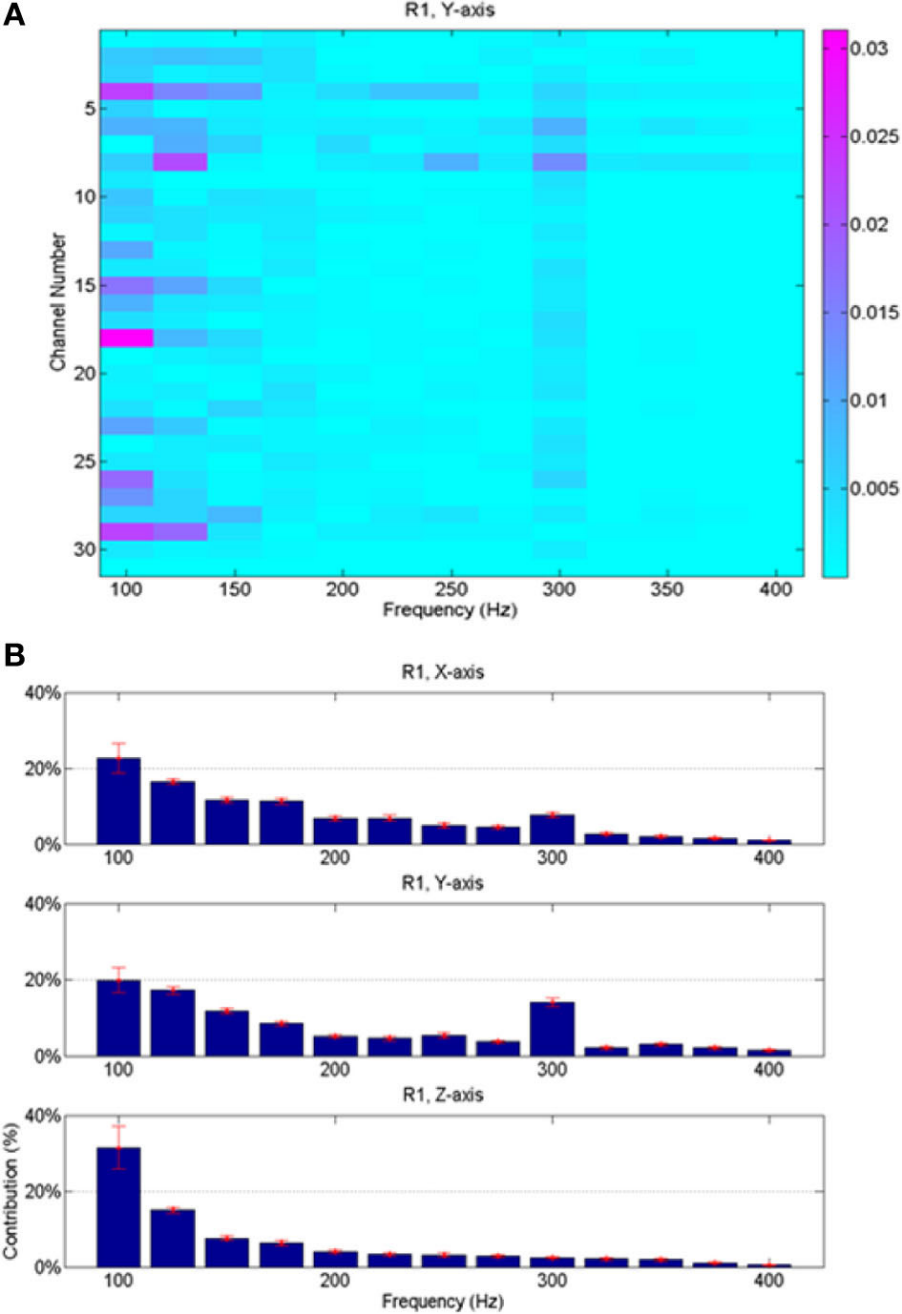
**Average neural contributions (from 23 trials shown in Figure 7) in different frequency bands to the reconstructed forces. (A)** Contributions from the contacts of the array vs. the frequencies; this plot is for the vertical force only. Each small rectangle indicates the average signal power across multiple trials at the corresponding frequency by the corresponding recording channel multiplied by its regression coefficient. Thus, the unit is the Newton. **(B)** Contributions from all contacts are lumped together and the mean and standard deviations (across all the trials in the session) are shown as a function of frequency for all three directions of the force. Highest contributions come from lower end of the spectrum, although there is a local maximum around 300 Hz for *X* and *Y* forces.

Since the neural signals were separated into various frequency bands (Figure 4), it was possible to do back projection and determine the frequency components and the neural channels that were selected more often than others by the regression algorithm. The relative contributions of various neural channels and frequencies are plotted in Figure 8 for rat R1. The middle bar plot in Figure 8B corresponds to the *y*-force map in Figure 8A with all the contact contributions lumped together to show relative power contributions in different frequency bands with means and standard deviations calculated across trials. The bar plots for the other two directions (*X* and *Z*) were produced from similar maps. It is interesting to note that lower frequencies contributed more to the predicted force in all three dimensions. There is a local maximum around 300 Hz in the *X* and *Y* axes. Frequency contributions were calculated as mean power across all contacts averaged in time, i.e., over trials, and then converted into percentage by taking the power in each frequency band and dividing it by the total power. The small standard deviation bars, which were calculated on the percent power over multiple trials, suggest that the frequency contributions are relatively stable across multiple trials and somewhat similar in all three directions in this session. We limited the analysis retrospectively to frequencies below 425 Hz since percent contributions above this frequency were negligibly small. Components below 75 Hz had to be disregarded due to movement artifacts and 60 Hz contamination.

### Spatial distribution of neural sources

**Figure 9:**
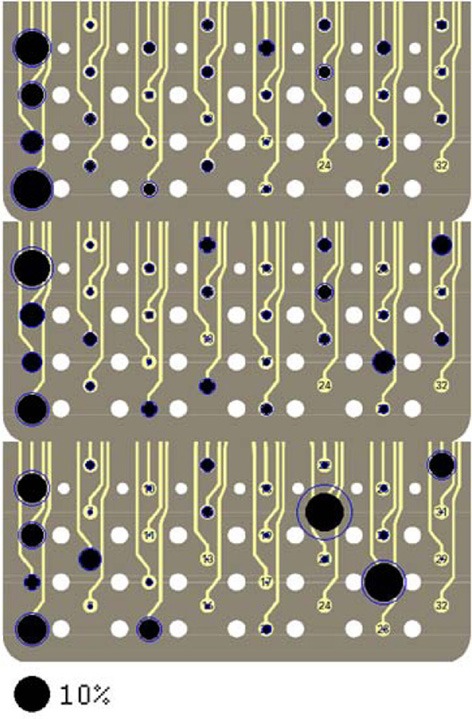
**Signal contributions from individual neural channels superimposed on three images of the electrode array in Rat 1.** Each image contains percent contributions for one direction of the forelimb force (*X*, *Y*, and *Z*, **top** to **bottom**). The sizes of the filled, black circles indicate average percent contributions across multiple trials in the session and the rings around the circles show the standard deviations. The large black circle on the bottom represents a contribution of 10%. Contacts 24 and 32 were not used.

The spatial organization of the neural channels (i.e., contacts) on the array was also determined by back projection. Figure 9 illustrates the signal strengths (in all frequencies) by each contact on the array for all directions of force in rat R1. Interestingly, certain contacts were selected much more frequently than others and these contacts are located mostly near the caudal end of the array (left side) for all three directions of force. No single contact dominates as a signal source and not all the contacts make a significant contribution. It is encouraging to note for a neural interface that the standard deviations (shown as rings around contacts) are not very large, which suggest spatial stability of the neural sources that are selected by the algorithm across multiple trials in this session.

### Group results

**Figure 10:**
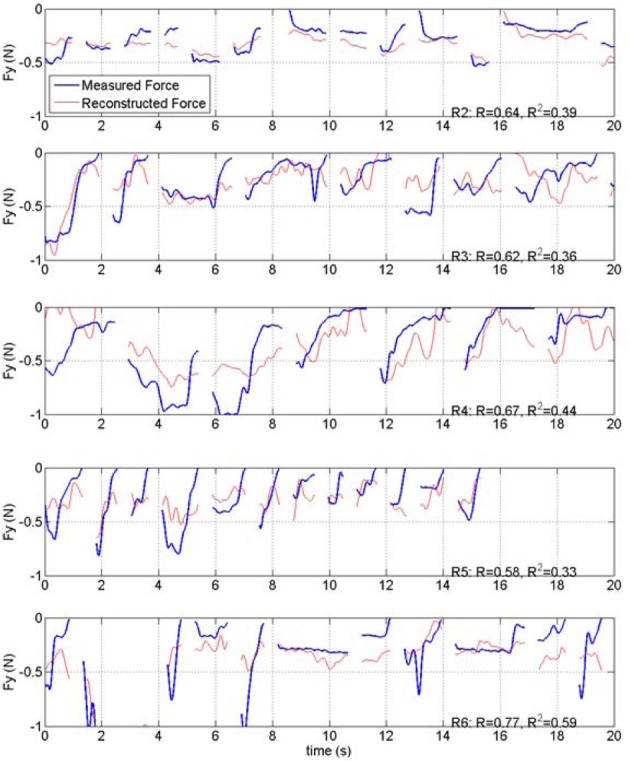
**Plot of measured and reconstructed forces in the test sets from rats 2, 3, 4, 5, and 6 of the study (R1 is shown in Figure 7).** Only the vertical forces are shown. Correlations and *R*^2^ values are indicated on each plot.

Force reconstruction plots (from the test sets) in the remaining rats of this study are shown in Figure 10. Only the vertical forces are plotted for brevity. In each implant, the vertical force amplitudes were predicted by the algorithm with a correlation coefficient that is above 0.58. The duration of the isometric lever holding and the force profiles during each trial were substantially different (Test lengths column in Table 1). Moreover, each animal employed somewhat different strategies in its lever pressing behavior, as observed in video records. Therefore, the force data profiles did not appear to be stereotypical and thereby allowing a large area of the parameter space to be visited.

**Figure 11:**
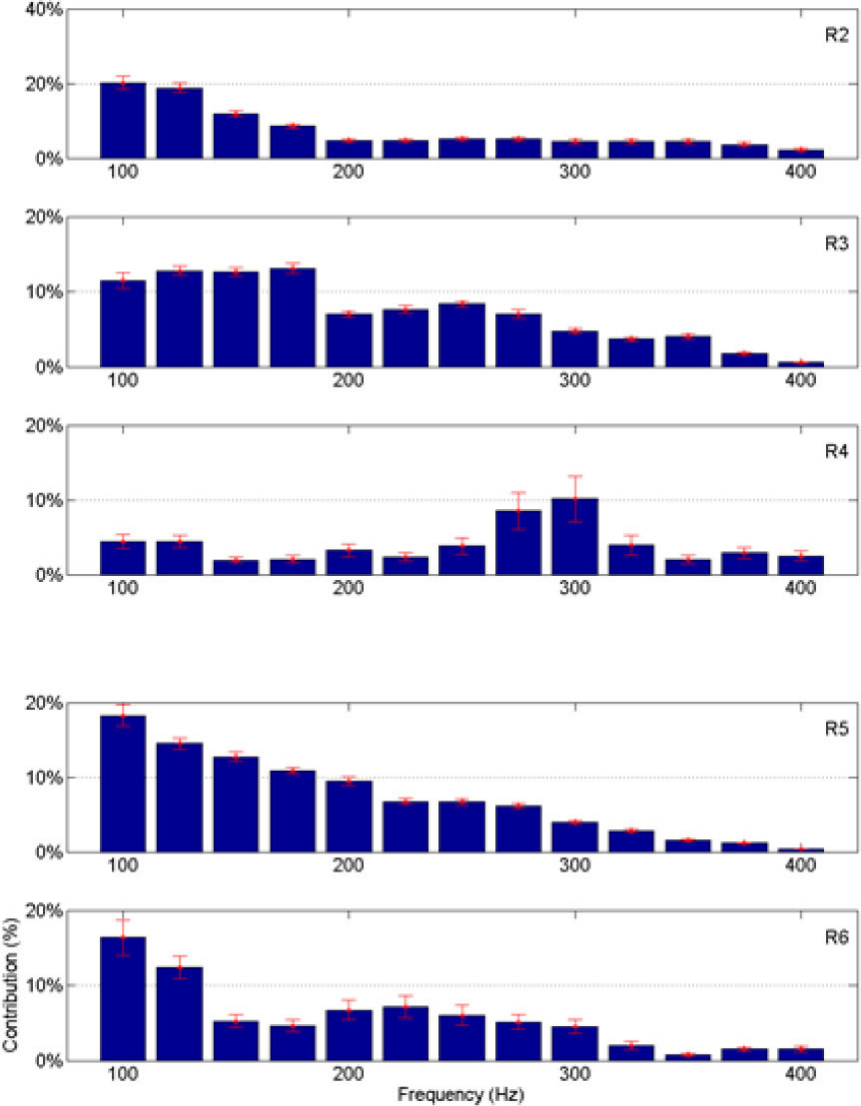
**Percent neural contributions to the vertical force (*Y*) in different frequency bands for the rats R2 through R6 (top to bottom)**.

The frequency band contributions to the vertical force predictions are shown in Figure 11 for all the remaining animals. The mean and standard deviations are calculated across all the trials in the test sets. As in Figure 8, most of the signal power comes from the lower frequencies, except in R4 where there is a peak around 300 Hz. Again, the standard deviations are small showing band limited signal contributions vary only slightly across multiple trials in each animal.

**Figure 12:**
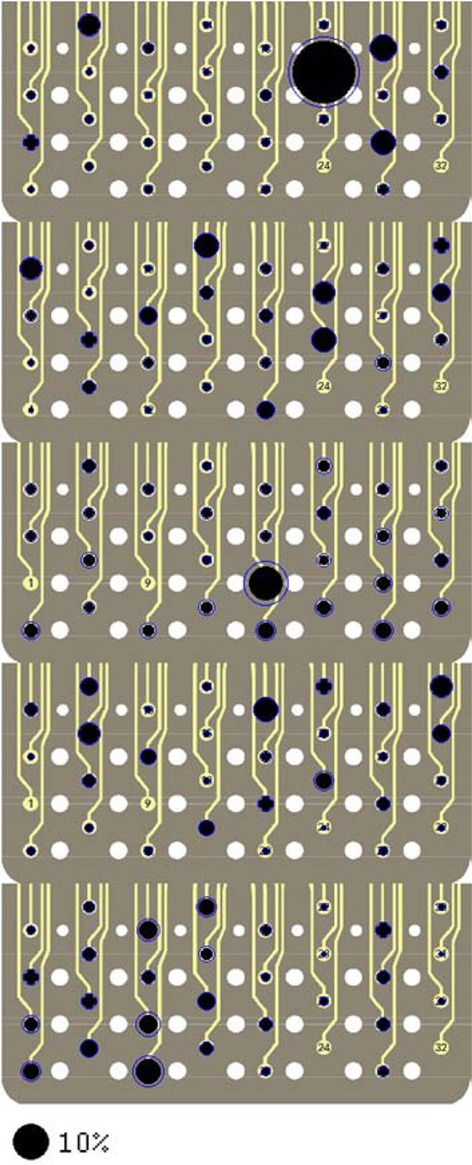
**Signal contributions to the vertical force (*Y*) from individual neural channels superimposed on an image of the electrode array for each of the rats R2 through R6 (top to bottom).** See Figure 9 caption for further explanation.

The spatial locations of the neural sources are depicted in Figure 12 for the remaining rats of this study. Only the vertical force maps are shown for brevity. Relatively small standard deviations, particularly for the contacts with large percent contributions, increase the confidence level in the reproducibility of the neural source locations within the spinal cord. The map does not reveal a preference in the dorsoventral direction that would point to a certain depth where most neural sources controlling the forelimb muscles might be located in the spinal cord cross-section. That is, the plots do not support the presence of somatotopic organization in the CST. The map looks very different in each animal, although some variation is expected since it is virtually impossible to implant the array in the same position in each animal and there are most probably some anatomical differences between the animals. In general, the contributions distribute across many contacts and even the largest contributions do not go above 10%, with one exception in R2.

### Statistics of reconstruction

Group statistics are summarized in Tables 1 and 2. The number of trials in the training and test sets are shown in the left most columns of Table 1. The best correlation and *R*^2^ values were obtained with R6, and the lowest coefficients with R5. Both measures were higher for the vertical force (*Y*) and the magnitude of the force vector (ABS) than that for the other two horizontal directions (*X* and *Z*) in all subjects. The vertical correlation varied between 0.58 and 0.77 with a mean ± STD of 0.66 ± 0.07 (*N* = 6). Similarly, the *R*^2^ value for the vertical force changed between 0.33 and 0.59 with mean ± STD of 0.43 ± 0.10 (*N* = 6). The *R*^2^ values are lower because this measure looks for an exact match between the actual and reconstructed forces, unlike the correlation coefficient which quantifies only the waveform similarity. Both measures for the ABS force were very close to that of the vertical force, presumably because the vertical force was the dominating component in the force vector. The last row in the table contains the mean values weighted by the number of trials in each test set. The weighted means are slightly higher because the largest test sets produced better predictions, especially in R5.

**Table 2:** **Group statistics of all trials**.

	**Training + Test R**					**Training + Test R2**			
	**TOTAL**	***X***	***Y***	***Z***	**ABS**	***X***	***Y***	***Z***	**ABS**
Mean	68.00	0.57	0.72	0.50	0.70	0.32	0.53	0.26	0.50
STD	29.47	0.13	0.10	0.09	0.10	0.14	0.14	0.09	0.15
Weighted		0.58	0.71	0.47	0.70	0.32	0.51	0.23	0.49

Correlation (R) and R^2^ statistics in all the trials (both training and test sets) lumped together from all rats to show that the performance of the algorithm was comparable (and not superior) to that of test sets.

The mean correlations and *R*^2^ values are presented in Table 2 from entire sessions including the training and test sets. These numbers illustrate that the regression algorithm performed only slightly better in the training set than it did in the test set, another evidence showing that the regression was not an over-fit to the data.

## Discussion

### Frequency analysis

Time-Frequency analysis (Cai et al., 2000) was invaluable in separating the neural signals that are most relevant to the forearm forces. Short Time Fourier Transform allowed multiple time-varying signals to be generated from a single physical channel. A single physical electrode can carry information from different neural sources in different frequency bands, and extraction of these sources into different channels was useful to improve the prediction. It is important to note that greater availability of signals required a larger training set. Additional trials were required to accurately estimate the extended set of coefficients.

The persistency of relative signal contributions to the force in various different frequency bands (Figures 8, 11) can be interpreted in different ways. This may be because the neural signals are composed of individual action potentials that have stereotypical shapes, which implies similar frequency components. However, the multi-unit neural signals usually occupy a frequency band starting around 300 Hz and reach up to a few kHz. The entire spectrum may have moved to lower frequencies in these recordings because of the small size spectra of the fibers in the rat CST. The fact that the frequency spectrum of the raw neural activity was reaching up to 1 kHz and even higher argues against this interpretation that the algorithm was selecting multi-unit signals. Individual action potentials were observed to last less than a millisecond in the raw data. The fundamental frequencies and harmonics of single spikes should be above 1 kHz. The large contributions below 200 Hz may be interpreted as local field potentials. This raises the question of possible contamination from the gray matter neurons into the recorded activity. However, the largest neural activities are not recorded from the most ventral contacts in Figure 12, as one would expect if the array is too close to the gray matter in the dorsal column. Therefore, the most plausible explanation is that the low frequency components represent the firing rate of axons within the white matter. Mean firing rates of individual neurons in the primate motor cortex peak around 50 pps during behavior (Grammont and Riehle, 2003), a much smaller value than the frequency components contained in a single action potential. Even smaller frequencies can be present in the signals due to modulation of neural spike rates in time. Unfortunately, components below 75 Hz had to be discarded in this study to ensure that 60 Hz contaminations were completely suppressed. It is possible that the frequencies selected by our algorithm are the harmonics of those slower signal components.

### Factors on regression success

Compared to brain-computer interfaces where single spike activities are used to predict the forelimb or arm kinematics, the spinal method should be able to access signal with much richer volitional content. Nonetheless, it may still not be possible to collect all the forelimb related activity from the CST because of the limited recording range of the electrode array. The dorsoventral extent of the electrode is approximately 300 μm and it matches the size of the CST in the rat. However, the majority of the fibers are smaller than 3.7 μm, as measured at the pyramids (Mediratta and Nicoll, 1983), (thus densely populated in a small cross sectional area of the dorsal column) and the number of recording contacts in the array is too small compared to the number of neural sources even if a small percentage of axons become active simultaneously at any time instant. This issue of under sampling the available neural sources can be resolved to a large extent with much denser arrays and smaller contacts.

Force prediction could also be limited by the fact that the RST activity was not accessed in this study. The RST plays an important role in voluntary movements both in rodents and primates (Jarratt and Hyland, 1999; Iwaniuk and Whishaw, 2000; Webb and Muir, 2003). The RST is also known to take over the function of an injured CST (Belhaj-Saif and Cheney, 2000). A second array implanted in the RST can demonstrate the predictive power of this tract in comparison to the CST in different behavioral contexts in future experiments.

Accounting for the neural and muscle activation delays had negligible effects on the success of reconstructions. This may be explained by small propagation delays in neural conduction and the fact that very fast changing force components were taken out by low-pass filtering. The myelinated descending fibers reach up to velocities ~19 m/s and the mean velocity is about 11.4 m/s (Mediratta and Nicoll, 1983), where the latter may be an overestimation in the cited study because of the tendency of electrical stimulations to recruit the larger and hence faster fibers first. Nevertheless, because the distance to the muscle is only in the order of centimeters, most of the delay can be attributed to muscle activation. The delay from the stimulation of cervical gray matter to the forelimb force initiation was less than 50 ms (unpublished results) in anesthetized rats in our laboratory. Because the forelimb forces did not contain very fast changes accounting for the neural conduction delay did not have a substantial effect on regression results.

### Recording electrodes

Two other types of electrodes were tested previously in our laboratory and the performance of flexible electrode arrays was deemed superior to both for this application. Utah type penetrating electrodes can generate severe neural damage in small animals due to their rigid substrate not conforming around the spinal cord (Rousche and Normann, 1998). Flexible electrode array also offered an additional benefit of recording from multiple sites in the same sagittal plane. The well-defined contact positions allowed a more uniform sampling of the CST activity in the mid-sagittal plane. Single wire electrodes had a tendency to disintegrate (Prasad and Sahin, 2006b) faster than the flexible electrode arrays even when wires were bundled within a silicone tube. Furthermore, the relative positions of wire electrodes were very difficult to control during implantation.

### Concerning a spinal cord computer interface

The long term objective of the study is to show the feasibility of extracting volitional components from the proximal side of the severed axons (at some distance from injury site) which were once serving the distal muscles. It should be noted that, for a spinal cord-computer interface, the targeted axons for recording in the proximal spinal cord are not only those that used to project to the upper limbs. Any neural component that is under volitional control can be utilized as a command signal in control of a prosthetic arm or other smart aids.

Needless to say, demyelination or degeneration of proximal axons would compromise the quality and information content of the targeted signals. Fortunately, the time course of retrograde degeneration in the lateral CST is much slower in humans than in rodents and a significant portion of the fibers is preserved even years after injury (Bronson et al., 1978; Fishman, 1987). Fishman (1987) reported from 12 patients post-mortem (carefully selected from a population of 343) who had an injury no lower than T-10 and the shortest survival time of two years from the time of trauma. Spinal cord sections within a few spinal segments of the injury were grossly depleted of CST axons, however, patients with high thoracic to low cervical lesions had normal appearing CST by the high cervical levels regardless of post-injury times. Also, in rats transected at T8, a majority of the axons stopped dying back four weeks after injury at about 2.5 mm from the site and at 16 weeks the mean distance at which terminal bulbs were found was only 4 mm (Seif et al., 2007). These reports in animals and human cadavers strongly suggest that the descending signals should be available for long term recordings within the cervical CST, although no reports of such recordings are found in the literature to our knowledge.

## Conclusions

This study demonstrated that volitional information contained in the CST about the forelimb forces can be accessed in the rat using flexible substrate array electrodes. The flexible MEAs may be a good choice to achieve a mechanically stable neural interface in the spinal cord. These results support the supposition that spinal cord-computer interfaces can eventually be built for subjects with spinal cord injury to operate manipulators with relatively little training.

### Conflict of interest statement

The authors declare that the research was conducted in the absence of any commercial or financial relationships that could be construed as a potential conflict of interest.

## References

[B1] Alstermark B. Ogawa J. Isa T. (2004). Lack of monosynaptic corticomotoneuronal EPSPs in rats: disynaptic EPSPs mediated via reticulospinal neurons and polysynaptic EPSPs via segmental interneurons. J. Neurophysiol. 91, 1832–1839 10.1152/jn.00820.2003 14602838

[B2] Baker S. N. Olivier E. Lemon R. N. (1997). Coherent oscillations in monkey motor cortex and hand muscle EMG show task-dependent modulation. J. Physiol. 501(Pt 1), 225–241 10.1111/j.1469-7793.1997.225bo.x 9175005 PMC1159515

[B3] Belhaj-Saif A. Cheney P. D. (2000). Plasticity in the distribution of the red nucleus output to forearm muscles after unilateral lesions of the pyramidal tract. J. Neurophysiol. 83, 3147–3153 10805709 10.1152/jn.2000.83.5.3147

[B4] Biran R. Martin D. C. Tresco P. A. (2005). Neuronal cell loss accompanies the brain tissue response to chronically implanted silicon microelectrode arrays. Exp. Neurol. 195, 115–126 10.1016/j.expneurol.2005.04.020 16045910

[B5] Bronson R. Gilles F. H. Hall J. Hedley-Whyte E. T. (1978). Long term post-traumatic retrograde corticospinal degeneration in man. Hum. Pathol. 9, 602–607 10.1016/S0046-8177(78)80143-9 711235

[B6] Cai L. Y. Wang Z. Z. Zhang H. H. (2000). [A surface EMG signal identification method based on short-time Fourier transform]. Zhongguo Yi Liao Qi Xie Za Zhi 24, 133–136 12583117

[B7] Carmel J. B. Kim S. Brus-Ramer M. Martin J. H. (2010). Feed-forward control of preshaping in the rat is mediated by the corticospinal tract. Eur. J. Neurosci. 32, 1678–1685 10.1111/j.1460-9568.2010.07440.x 21044175 PMC3058632

[B8] Casale E. J. Light A. R. Rustioni A. (1988). Direct projection of the corticospinal tract to the superficial laminae of the spinal cord in the rat. J. Comp. Neurol. 278, 275–286 10.1002/cne.902780210 3230165

[B9] Cheney P. D. Mewes K. Fetz E. E. (1988). Encoding of motor parameters by corticomotoneuronal (CM) and rubromotoneuronal (RM) cells producing postspike facilitation of forelimb muscles in the behaving monkey. Behav. Brain Res. 28, 181–191 10.1016/0166-4328(88)90095-2 3132935

[B10] Conway B. A. Halliday D. M. Farmer S. F. Shahani U. Maas P. Weir A. I. (1995). Synchronization between motor cortex and spinal motoneuronal pool during the performance of a maintained motor task in man. J. Physiol. 489(Pt 3), 917–924 8788955 10.1113/jphysiol.1995.sp021104 PMC1156860

[B11] Dunkerley G. B. Duncan D. (1969). A light and electron microscopic study of the normal and the degenerating corticospinal tract in the rat. J. Comp. Neurol. 137, 155–183 10.1002/cne.901370204 5821843

[B12] Entakli J. Bonnard M. Chen S. Berton E. De Graaf J. B. (2013). TMS reveals a direct influence of spinal projections from human SMAp on precise force production. Eur. J. Neurosci. 39, 132–140 10.1111/ejn.12392 24164635

[B13] Fishman P. S. (1987). Retrograde changes in the corticospinal tract of posttraumatic paraplegics. Arch. Neurol. 44, 1082–1084 10.1001/archneur.1987.00520220078021 3632383

[B14] Gorgels T. G. (1990). A quantitative analysis of axon outgrowth, axon loss, and myelination in the rat pyramidal tract. Brain Res. Dev. Brain Res. 54, 51–61 10.1016/0165-3806(90)90064-6 2364545

[B15] Grammont F. Riehle A. (2003). Spike synchronization and firing rate in a population of motor cortical neurons in relation to movement direction and reaction time. Biol. Cybern. 88, 360–373 10.1007/s00422-002-0385-3 12750898

[B16] Guo Y. Mesut S. Foulds R. A. Adamovich S. V. (2013). Corticospinal signals recorded with MEAs can predict the volitional forearm forces in rats. Conf. Proc. IEEE Eng. Med. Biol. Soc. 2013, 1984–1987 10.1109/EMBC.2013.6609918 24110105 PMC4063445

[B17] Iwaniuk A. N. Whishaw I. Q. (2000). On the origin of skilled forelimb movements. Trends Neurosci. 23, 372–376 10.1016/S0166-2236(00)01618-0 10906801

[B18] Jarratt H. Hyland B. (1999). Neuronal activity in rat red nucleus during forelimb reach-to-grasp movements. Neuroscience 88, 629–642 10.1016/S0306-4522(98)00227-9 10197781

[B19] Kubanek J. Miller K. J. Ojemann J. G. Wolpaw J. R. Schalk G. (2009). Decoding flexion of individual fingers using electrocorticographic signals in humans. J. Neural Eng. 6:066001 10.1088/1741-2560/6/6/066001 19794237 PMC3664231

[B20] Leenen L. P. Meek J. Posthuma P. R. Nieuwenhuys R. (1985). A detailed morphometrical analysis of the pyramidal tract of the rat. Brain Res. 359, 65–80 10.1016/0006-8993(85)91413-1 4075163

[B21] Leenen L. P. Meek J. Posthuma P. R. Nieuwenuys R. (1989). Differences in the fiber composition of the pyramidal tract in two- and 14-month-old rats. Neuroscience 28, 635–643 10.1016/0306-4522(89)90010-9 2710336

[B22] Lemon R. N. Mantel G. W. Muir R. B. (1986). Corticospinal facilitation of hand muscles during voluntary movement in the conscious monkey. J Physiol. 381, 497–527 3625543 10.1113/jphysiol.1986.sp016341 PMC1182993

[B23] Liang F. Y. Moret V. Wiesendanger M. Rouiller E. M. (1991). Corticomotoneuronal connections in the rat: evidence from double-labeling of motoneurons and corticospinal axon arborizations. J. Comp. Neurol. 311, 356–366 10.1002/cne.903110306 1720143

[B24] Mediratta N. K. Nicoll J. A. (1983). Conduction velocities of corticospinal axons in the rat studied by recording cortical antidromic responses. J. Physiol. 336, 545–561 6875920 10.1113/jphysiol.1983.sp014597 PMC1198984

[B25] Murray H. M. Gurule M. E. (1979). Origin of the rubrospinal tract of the rat. Neurosci. Lett. 14, 19–23 10.1016/0304-3940(79)95337-0 530486

[B26] Nishimura Y. Perlmutter S. I. Fetz E. E. (2013). Restoration of upper limb movement via artificial corticospinal and musculospinal connections in a monkey with spinal cord injury. Front. Neural Circuits 7:57 10.3389/fncir.2013.00057 23596396 PMC3622884

[B27] Omlor W. Patino L. Hepp-Reymond M. C. Kristeva R. (2007). Gamma-range corticomuscular coherence during dynamic force output. Neuroimage 34, 1191–1198 10.1016/j.neuroimage.2006.10.018 17182258

[B28] Park M. C. Belhaj-Saif A. Cheney P. D. (2004). Properties of primary motor cortex output to forelimb muscles in rhesus macaques. J. Neurophysiol. 92, 2968–2984 10.1152/jn.00649.2003 15163675

[B29] Perge J. A. Homer M. L. Malik W. Q. Cash S. Eskandar E. Friehs G. (2013). Intra-day signal instabilities affect decoding performance in an intracortical neural interface system. J. Neural Eng. 10:036004 10.1088/1741-2560/10/3/036004 23574741 PMC3693851

[B30] Prasad A. Sahin M. (2006a). Multi-channel recordings of the motor activity from the spinal cord of behaving rats. Conf. Proc. IEEE Eng. Med. Biol. Soc. 1, 2288–2291 10.1109/IEMBS.2006.260854 17946950 PMC3068858

[B31] Prasad A. Sahin M. (2006b). Extraction of motor activity from the cervical spinal cord of behaving rats. J. Neural Eng. 3, 287–292 10.1088/1741-2560/3/4/005 17124332 PMC2396534

[B32] Rousche P. J. Normann R. A. (1998). Chronic recording capability of the Utah Intracortical Electrode Array in cat sensory cortex. J. Neurosci. Methods 82, 1–15 10.1016/S0165-0270(98)00031-4 10223510

[B33] Schalk G. Miller K. J. Anderson N. R. Wilson J. A. Smyth M. D. Ojemann J. G. (2008). Two-dimensional movement control using electrocorticographic signals in humans. J. Neural Eng. 5, 75–84 10.1088/1741-2560/5/1/008 18310813 PMC2744037

[B34] Seif G. I. Nomura H. Tator C. H. (2007). Retrograde axonal degeneration “dieback” in the corticospinal tract after transection injury of the rat spinal cord: a confocal microscopy study. J. Neurotrauma 24, 1513–1528 10.1089/neu.2007.0323 17892412

[B35] Shin D. Watanabe H. Kambara H. Nambu A. Isa T. Nishimura Y. (2012). Prediction of muscle activities from electrocorticograms in primary motor cortex of primates. PLoS ONE 7:e47992 10.1371/journal.pone.0047992 23110153 PMC3480494

[B36] ten Donkelaar H. J. (1988). Evolution of the red nucleus and rubrospinal tract. Behav. Brain Res. 28, 9–20 10.1016/0166-4328(88)90072-1 3289562

[B37] Webb A. A. Muir G. D. (2003). Unilateral dorsal column and rubrospinal tract injuries affect overground locomotion in the unrestrained rat. Eur. J. Neurosci. 18, 412–422 10.1046/j.1460-9568.2003.02768.x 12887423

[B38] Whishaw I. Q. Gorny B. (1996). Does the red nucleus provide the tonic support against which fractionated movements occur? A study on forepaw movements used in skilled reaching by the rat. Behav. Brain Res. 74, 79–90 10.1016/0166-4328(95)00161-1 8851917

[B39] Whishaw I. Q. Gorny B. Sarna J. (1998). Paw and limb use in skilled and spontaneous reaching after pyramidal tract, red nucleus and combined lesions in the rat: behavioral and anatomical dissociations. Behav. Brain Res. 93, 167–183 10.1016/S0166-4328(97)00152-6 9659998

